# The role for saxagliptin within the management of type 2 diabetes mellitus: an update from the 2010 European Association for the Study of Diabetes (EASD) 46th annual meeting and the American Diabetes Association (ADA) 70th scientific session

**DOI:** 10.1186/1758-5996-2-69

**Published:** 2010-12-15

**Authors:** Pablo J Aschner

**Affiliations:** 1 Javeriana University, Avenida 15 No 124-47 Of 409, Bogotá, Colombia; 2 San Ignacio University Hospital, San Ignacio, Colombia

## Abstract

Saxagliptin is a potent, selective DPP4 inhibitor. Highlights from abstracts presented at the 2010 meetings of the European Association for the Study of Diabetes and the American Diabetes Association include studies and analyses that shed light on the promising role for saxagliptin within the management of type 2 diabetes mellitus. Data show that saxagliptin combination therapy improves HbA_1c _levels compared with placebo, particularly in patients with high HbA_1c _at baseline, long duration of disease, low baseline creatinine clearance, and low homeostasis model assessment 2 β-cell function at baseline. These efficacy benefits are achieved without any increase in hypoglycemia or other adverse events. The study results also show that the saxagliptin plus metformin combination is a good candidate for initial therapy in drug-naïve patients treated for as long as 72 weeks. Survey data presented confirm that hypoglycemia (and fear of hypoglycemia) is a barrier to patients' acceptance of diabetes treatment, limiting its efficacy. Therefore, therapies such as saxagliptin that have a low risk of hypoglycemia may be more acceptable to patients in helping them to achieve glycemic control and to optimize their quality of life. In patients with renal impairment, for whom metformin is contraindicated, saxagliptin monotherapy is a promising option for antidiabetic management as, when given at a reduced dose, it is well-tolerated with a safety profile similar to that of placebo.

## Introduction

The dipeptidyl peptidase-4 (DPP4) inhibitors are a novel class of antidiabetic drugs with a good safety profile and which are now being considered in some algorithms for the treatment of type 2 diabetes mellitus (T2DM). As evidence accumulates, it is likely that they will occupy a more predominant role. We present here some of the key data on saxagliptin (SAXA) - a potent, selective DPP4 inhibitor - derived from abstracts presented at the 2010 meetings of the American Diabetes Association (ADA) and the European Association for the Study of Diabetes (EASD).

### Efficacy of saxagliptin as an add-on therapy

At EASD, Allen et al presented a pooled analysis of Phase III trials comparing SAXA with placebo as an add-on therapy to metformin (MET), sulphonylureas (SU) or thiazolidinedione (TZD) [1]. Patients with T2DM recruited into the studies were aged >18 years, with HbA_1c _≥7%. The aim of the analysis was to evaluate any potentially important relationships between patient characteristics at baseline and the efficacy of SAXA 5 mg/day over a 24-week period. The following subgroups were defined according to baseline characteristics: gender (male, female); body mass index (BMI; <30, ≥30); age (< 65, ≥65 years); T2DM duration (≤1.5, ≤3, >3- < 5, ≥5, ≥10 years); creatinine clearance (≤0.8, >0.8 mL/s); homeostasis model assessment 2 β-cell function (HOMA-2B; ≤ and >58.6; baseline study median). The efficacy of SAXA (mean change from baseline in HbA_1c _versus placebo at week 24) was determined for each subgroup category. The results demonstrate that HbA_1c _lowering from baseline was greater with SAXA than with placebo. The decreases in HbA_1c _ranged from -0.52% to -0.93% across the diverse demographic and T2DM subgroups, including gender, BMI, age, and renal function. There was a trend toward larger mean HbA_1c _decreases in patients with longer duration of T2DM (≥5 years), lower baseline creatinine clearance, and lower baseline HOMA-2B [1].

The same group also analyzed the relationship between the efficacy of SAXA and baseline HbA_1c _in the Phase III trial pooled results [2]. As expected from previous metaregression analyses of trials with various oral antidiabetic drugs, the reductions in HbA_1c _were greater in patient subgroups with higher HbA_1c _levels at baseline. In those who had HbA_1c _8.6-8.9% and ≥9%, the mean placebo-corrected HbA_1c _reductions were -1% and -0.86%, respectively. Among patients who were close to target HbA_1c _at baseline (i.e. <7.5%), more than 50% achieved target HbA_1c _< 7% without associated hypoglycemic episodes [2]. Other adverse events were not reported within the abstract.

### Saxagliptin has similar efficacy without increasing hypoglycemia when compared with other insulin secretagogues

The ability of SAXA to lower HbA_1c _without increasing hypoglycemic episodes is due to its glucose-dependent effect and it is one of the main advantages of incretins when compared with other insulin secretagogues such as SU. Göke et al at tested this hypothesis in a 52-week multicenter, randomized, double-blind, noninferiority, Phase IIIb trial comparing the efficacy and safety of adding SAXA 5 mg/d or glipizide (GPZ) to MET in T2DM patients inadequately controlled on MET with an HbA_1c _> 6.5%-10% and on a stable MET dose ≥1500 mg/day. GPZ was uptitrated as needed from 5 to 20 mg/day (mean dose 14.7 mg/day; 51% received 20 mg/day) [3]. The results, which were presented at ADA, show that the efficacy of SAXA is noninferior to GPZ when added to MET, but treatment with SAXA was associated with significantly fewer hypoglycemic events (3.0% vs 36.3%; p < 0.0001) and a divergent favourable impact on body weight (mean change -1.1 vs + 1.1 kg; p < 0.0001). Treatment-related adverse events were less common with SAXA compared with GPZ (9.8% vs 31.2%). Excluding hypoglycemia, the proportion of patients experiencing adverse events was similar in the two groups (56.8% SAXA vs 54.9% GPZ) and discontinuation rates due to adverse events were also similar between groups (4%) [3].

### Saxagliptin plus metformin as initial therapy in drug-naïve patients

Perhaps one of the most interesting uses for the combination of a DPP4 inhibitor with MET may be as initial therapy for T2DM, due to the fact that the former would not add any titration problems nor adverse effects when combined with MET. At ADA Pfützner et al presented the long-term results of a multicenter, randomized, double-blind, active-controlled study, which had a 24-week short-term period (primary) followed by a 52-week long-term extension (total 76 weeks) to assess long-term efficacy and tolerability of initial combination therapy with SAXA plus MET versus SAXA or MET alone in drug-naïve patients with T2DM and elevated HbA_1c _(mean baseline HbA_1c _9.5%) [4]. SAXA was tested in combination at doses of 5 and 10 mg but the results were very similar. At week 76, reduction from baseline HbA_1c _was greater in the SAXA plus MET groups compared with the monotherapy groups (- 2.3% vs -1.6% with SAXA alone and -1.8% with MET alone) and the proportion of patients reaching target HbA_1c _< 7% was also greater (51% vs 25% and 35%, respectively). Incidence of adverse events was almost identical across groups (SAXA 10 mg plus MET 68.4%, SAXA plus placebo 66.3%, MET plus placebo 68.3%). In addition, the SAXA plus MET combination showed no increase in hypoglycemia compared with either monotherapy [4].

### Pharmacokinetic analyses

In the basic field, Zhang et al presented an abstract at ADA characterizing the pharmacokinetics of SAXA and its major active metabolite, 5-hydroxy SAXA, in healthy subjects and in patients with T2DM, in order to quantify the impact of subject-specific covariates on exposure and support the dosing recommendation in patients with T2DM, as well as its use in special populations [5]. The pharmacokinetics of SAXA and 5-hydroxy SAXA can be adequately described by a two-compartment linear model with no time-variant parameters, and were similar in healthy subjects and in patients with T2DM. There was no appreciable accumulation after once-daily dosing. Renal function (estimated by creatinine clearance) was identified as a significant covariate on the apparent clearance of SAXA and 5-hydroxy SAXA from the central compartment. No pharmacokinetic parameters were affected significantly by gender, body weight, age, or race. Results from exposure modeling of SAXA and 5-hydroxy SAXA in healthy subjects and in T2DM patients support once-daily dosing of SAXA without dosage adjustment on the basis of body weight, gender, age, or race alone, but suggest renal function should be taken into consideration when assessing SAXA dosage [5].

### Monotherapy in patients with renal impairment

To explore the clinical use of SAXA in patients with renal impairment, a multicenter, randomized, double-blind, parallel-group, placebo-controlled 12-week study conducted to the assess the efficacy and safety of SAXA 2.5 mg/day in adult patients with T2DM, HbA_1c _7%-11%, and moderate (estimated creatinine clearance ≥0.50 to <0.84 mL/s) to severe (creatinine clearance <0.50 mL/s) renal impairment, including end-stage renal disease (ESRD) with hemodialysis [6]. Patients (96%) continued background oral antidiabetic (32%) and/or insulin (74%) therapy as prescribed. The results, which were presented by Nowicki et al at ADA, showed that the overall mean reduction in HbA_1c _from baseline to week 12 for patients with renal impairment was significantly greater with SAXA versus placebo. Subgroup results by baseline renal impairment category showed numerically greater reductions in HbA_1c _with SAXA versus placebo in patients with moderate or severe renal impairment, while the HbA_1c _reduction in patients with ESRD on hemodialysis was similar between the treatment groups. SAXA was well tolerated, with a safety profile similar to placebo (adverse events were experienced by 57.6% of patients in the SAXA group, compared with 54.1% in the placebo group; hypoglycemia was reported in 20.0% of SAXA patients vs 22.4% of placebo patients) [6]. Based on these results, SAXA could also be considered as the antidiabetic drug in patients with moderate or severe renal impairment, where insulin is usually the drug of choice and MET is contraindicated, although it is not recommended for patients with ESRD on hemodialysis.

### β-cell preservation

Incretins and particularly glucagon-like peptide-1 (GLP-1) have a very promising effect on β-cell mass by inhibiting apoptosis and even stimulating β-cell formation from precursor cells. This has been demonstrated *in vitro *and *in vivo *in rodents. At EASD Poucher et al presented an experiment comparing the effect of SAXA and sitagliptin (SITA) - which inhibit DPP4 and reduce its deactivation of GLP-1 - on the preservation of pancreatic β-cell mass in high fat-fed streptozocin (STZ)-treated mice [7]. Glycemic control was determined by oral glucose tolerance tests and fasting blood glucose 3 weeks and 36 days, respectively, following STZ treatment. Terminal formalin-fixed, paraffin-embedded samples, taken 36 days after initiation of treatment, were analyzed for β-cell mass using automated imaging and analysis systems. In animals treated post-STZ, HbA_1c _levels compared with vehicle dosing (6.8%) were significantly lower with either SAXA (6.1%; p < 0.01) or SITA 6.3% (p < 0.05). β-cell mass was also significantly greater with SAXA (0.28 mg) and with SITA (0.42 mg) than with vehicle (p < 0.05 for both). Overall, both DPP4 inhibitors showed similar improvements in glycemic control and β-cell mass preservation in the high fat-fed, STZ mouse model of pancreatic β-cell degeneration [7]. This had been already shown with SITA but it was demonstrated for the first time with SAXA.

### Advantages of reduced hypoglycemia for patients

There were two additional abstracts presented at EASD that highlight the advantage of treatments such as SAXA that do not increase hypoglycemia: both reported the results of the PANORAMA study, a large pan- European cross-sectional survey (NCT00916513) of patients with T2DM treated with glucose-lowering therapies (either oral hypoglycemic agents [OHAs] or injectables - insulin and GLP-1 receptor analogues - with or without OHAs) [8,9]. Eligible patients were ≥40 years of age with a diagnosis of T2DM for >1 year prior to study entry and an available medical record at the clinic of >1 year. The analyses aimed to assess treatment satisfaction, quality of life, and the proportion of patients achieving an HbA_1c _< 7%. One of the abstracts reported recent data from nine countries on glycemic control in patients with T2DM in relation to treatment patterns. They found that although the level of glycemic control in Europe may be improving, there is still a gap between current management and optimal treatment of patients with T2DM [8]. The failure to reach target was greater as treatment was intensified, possibly due a longer duration of T2DM and to the concern over hypoglycemia. In fact, the authors demonstrate that the occurrence of both severe and nonsevere hypoglycemia in T2DM is associated with a greater negative impact of diabetes on quality of life, less treatment satisfaction, and greater fear of hypoglycemia, which may be an important barrier to optimal glycemic control in some patients [9].

## Conclusion

Abstracts presented at the ADA and EASD 2010 meetings show that SAXA is a potent, selective DPP4 inhibitor, which can be given once a day as an add-on therapy to patients on MET, SU, or TZD. Being an insulin secretagogue, it is as effective as an SU but without significant hypoglycemia and no weight gain. It is particularly effective in combination with MET as initial therapy and also well tolerated across different patient profiles, including patients with renal impairment receiving a reduced dose. DPP4 inhibitors may have a potential role in preserving β-cell function, although this has to be demonstrated in humans.

## List of abbreviations used

ADA: American Diabetes Association; DPP4 inhibitors: dipeptidyl peptidase-4 (DPP4) inhibitors; EASD: European Association for the Study of Diabetes; EASD: end-stage renal disease; GLP-1: glucagon-like peptide; GPZ: glipizide; HOMA-2B: homeostasis model assessment 2 β-cell function; MET: metformin; SAXA: saxagliptin; STZ: streptozotocin; SU: sulphonylureas; TZD: thiazolidinedione; T2DM: type 2 diabetes mellitus.

## Competing interests

The author declares to have received honoraria as lecturer and scientific advisor for AstraZeneca, MSD, Novartis, and Sanofi Aventis.

## Authors' contributions

PA reviewed the meeting abstracts and prepared the commentary.

## References

[B1] Allen E Donovan M Berglind N Maheux P Efficacy of saxagliptin according to patient baseline characteristics: a pooled analysis of three add-on pivotal randomised phase 3 clinical trials EASD 2010 826-P

[B2] Maheux P Donovan M Allen E Berglind N Bouzamondo H Efficacy of saxagliptin in relation to baseline HbA1c in a pooled analysis of three add-on pivotal randomised phase 3 clinical trials EASD 2010 825-P

[B3] Göke B Gallwitz B Eriksson J Hellqvist A Gause-Nilsson I Saxagliptin is non-inferior to glipizide when added to metformin in patients with type 2 diabetes mellitus ADA 2010 578-P

[B4] Pfützner A Paz-Pacheo E Berglind N Allen E Frederich B Chen B Saxagliptin initial combination with metformin provides sustained glycemic control and is well tolerated in patients with type 2 diabetes: 76-week results ADA 2010 64-OR

[B5] Zhang L Boulton DW Pfister M Exposure modeling of Saxagliptin and 5-Hydroxy Saxagliptin in healthy subjects and in patients with type 2 diabetes to support Saxagliptin dosing recommendations ADA 2010 675-P

[B6] Nowicki M Rychlik I Haller H Warren ML Suchower L Gausenilsson I Saxagliptin improves glycemic control and is well tolerated in patients with type 2 diabetes mellitus (T2DM) and renal impairment compared with placebo ADA 2010 550-P

[B7] Poucher SM Francis J Vickers S Cheetham S Birmingham G Dickinson K Zinker B Bellamine A Kirby M Preservation of pancreatic beta cell mass in high fat-fed STZ treated mice by the Dipeptidyl peptidase-4 inhibitors saxagliptin and sitagliptin EASD 2010 567-P

[B8] de Pablos-Velasco P Bradley C Eschwège E Gönder-Frederick L Parhofer K Vandenberghe H Simon D The PANORAMA pan-European survey: glycaemic control and treatment patterns in patients with type 2 diabetes EASD 2010 1012-P

[B9] Bradley C Eschwège E de Pablos-Velasco P Parhofer K Simon D Tafalla M Pascual E Gönder-Frederick L The PANORAMA pan-European Survey: Impact of severe and non-severe hypoglycaemia on quality of life and other patient reported outcomes in patients with type 2 diabetes EASD 2010 580-P

